# EEG Based Cortico-Muscular Connectivity During Standing Early Post Stroke

**DOI:** 10.1109/EMBC40787.2023.10341014

**Published:** 2023-07

**Authors:** Michael Glassen, Gregory Ames, Guang Yue, Karen J. Nolan, Soha Saleh

**Affiliations:** 1 Michael Glassen, Guang Yue, Karen Nolan and Soha Saleh are with Rutgers University, Newark, NJ 07107 USA; 2 Greg Ames, Guang Yue, and Karen J Nolan are with Kessler Foundation, West Orange, NJ 07052 US

## Abstract

In this exploratory study we studied brain activation and corticomuscular connectivity during standing in healthy individuals and persons with stroke within 40 days of cerebrovascular accident (CVA). EEG and EMG data were acquired during standing and analysis showed a trend of higher EEG power (hyper activation) in the stroke group. Direct corticomuscular connectivity between sensorimotor cortices and contralateral lower extremity muscles showed lower connectivity between affected motor, premotor, and sensory cortices, and contralateral lower extremity peripheral muscles with moderate effect size. The preliminary data in this paper suggest re-organization in left sensorimotor cortex role in controlling contralateral lower extremity muscles during standing. Correlational analysis in stroke group within 40 days of CVA showed a relationship between higher corticomuscular connectivity and better scores on balance assessments.

## I. Introduction

Stroke is the leading cause of serious long-term disability in the United States, where over 1 million Americans experience functional limitations in activities of daily living because of stroke [1]. While major advances have been made to treat patients post-stroke, the majority of survivors have residual mobility challenges [1], [2] including change in motor strategies [3] and balance deficits [4]. There have been major advances made in the treatment of patients post-stroke, but the majority of survivors are left with residual mobility deficits [2], [5]. It is possible for the central nervous system (CNS) to reorganize itself after injury, and this potential for restructuring is related to the intensity and quality of the performed motor activity during rehabilitative training [6]. There is commonly a delay in administering high dosage walking training after stroke, sometimes for weeks, causing maladaptive brain plasticity which is difficult to renormalize. The acute stage within 40 days post stroke is critical to recovery, as during this time the injured CNS is highly responsive to repetitive goal-directed physical activities. In an ongoing study at Kessler Foundation, we compare the effects of progressive high-dose exoskeleton assisted walking training versus standard of care (SOC) administered during the acute phase of stroke on functional recovery and neuromuscular activation. We investigate whether the reorganization in cortico-muscular connectivity (CMC) is an adaptation to stroke or a re-normalization to connectivity before stroke. A major outcome in this study is exploring reorganization in brain connectivity and corticomuscular connectivity after stroke and over the course of recovery. This paper will present the methodologies used to study CMC during standing and preliminary results in stroke and healthy control (HC) participants. Previous research showed relationship between symmetry in EEG power during sitting and standing and balance dysfunction in stroke survivors [7]. While research specifically focusing on corticomuscular connectivity during standing after stroke is limited, some studies have examined corticomuscular interactions during other motor tasks in stroke survivors. These studies have shown altered corticomuscular coherence patterns and reduced corticomuscular connectivity in stroke survivors, mainly in acute phase [8], compared to healthy individuals. In this paper, we investigate EEG power during standing, in addition to connectivity between selected regions in the sensorimotor cortex and contralateral lower extremity muscles. CMC connectivity was also correlated to clinical measures obtained at baseline.

## II. Methodology

### A. Participants

Nine stroke patients were recruited from the inpatient population at the Kessler Institute of Rehabilitation (KIR) and completed baseline measures. Subjects were enrolled in the study within 7 to 30 days post stroke and were randomly assigned to one of two training groups. Four age and gender-matched healthy controls were enrolled to serve as “gold standard” of within-brain and brain-to-muscle connectivity for evaluation of the connectivity re-normalization in the stroke patients following intervention. All participants provided informed consent approved by Kessler Foundation Institution Review Board.

### B. Data Collection Method

Data was collected at baseline and after 10 weeks of gait training (1 hour a day, 3 days a week). In this paper, we present both baseline and post-training data (only six stroke subjects completed post-training evaluation). During data collection, subjects were asked to complete a standing task while using electroencephalography (EEG) to record brain activity and electromyography (EMG) to record lower extremity muscle activity. Subjects were outfitted with reflective markers and motion data were recorded using Motive motion capture software combined with Optitrack cameras (NaturalPoint INC, Corvallis, OR USA). EEG, EMG and motion capture software were all synced using TTL pulses. EMG data was acquired from gastrocnemius muscle, soleus, and tibialis anterior muscle. **Standing Task:** Data were acquired during 6 trials of 15 seconds standing.

**Clinical Measures:** A trained physical therapist administered the Berg Balance Test for each subject before the beginning of training. The Movendo balance platform (Movendo Technology, Genova, Italy) was also used to perform a Silver Index assessment of fall and assessments of displacement in center of pressure in response to balance platform perturbations in standing conditions. EEG and EMG data were not measured during these perturbations.

**Data Processing:** EMG data was collected using Delsys Trigno wireless EMG sensors (Delsys INC, Natick, MA, USA), and band-pass filtered (4–400Hz) in MATLAB using a 2^nd^ order Butterworth filter. EEG data was acquired with the actichamp+ amplifier and 64 channel gel-based electrode cap (Brain Vision LLC, Morrisville, NC, USA). Electrodes were placed using the 10/20 international system, with the reference channel placed at Cz channel. EEG data were preprocessed by session, utilizing custom scripts written for the EEGLAB Matlab toolbox. Using the cleanrawdata EEGLAB plugin, EEG channels containing artifacts or flat periods were marked for removal and Artifact Subspace Reconstruction (ASR) was used to remove noise from the data. An average reference was applied to all remaining channels, and the Prep plugin for EEGLAB was used to remove 60 Hz line noise. The 15 seconds of standing data from each trail were grouped together and epoched (split into evenly spaced 1-second epochs). Epochs containing artifacts were deleted manually through visual inspection. Independent Component Analysis (ICA) was applied to epoched data and components deemed likely to represent artifacts (muscle activity, eye blinks, channel noise) were rejected with the help of the EEGLAB plugin ICLabel.

Preprocessed and epoched EEG data was then imported into the Fieldtrip toolbox for MATLAB. Electrode locations used during collection were then projected onto the basic head model included in Fieldtrip, created from the Colin27 MRI. A lead field source model was computed using this head model to a 4 mm resolution. Using linearly constrained minimum variance (LCMV) beamformer source analysis techniques, source data was calculated for the entire lead field (~30,000 points) on a per-trial basis, and then averaged together within selected regions of interest chosen from the Brainnetome atlas [9]. After combining the trial by source data with the processed EMG signals, a time-invariant Multivariate Autoregressive Model (MVAR) was created, with an optimal model order between 5 and 20 was chosen using the SIFT EEGLAB plugin. Normalized Directed Transfer Function connectivity(nDTF), a linear connectivity measure shown to perform well even on non-linear data such as EEG [11], was calculated between selected ROIs in the Brainnetome atlas and EMG channels using this model and functions provided by Fieldtrip.

EEG power and DTF connectivity within the beta band (12–30 Hz) were estimated for the following list of selected ROIs within the sensorimotor cortex: left and right parts of the primary motor cortices associated with trunk and lower extremity (lM1 and rM1), left and right parietal cortices (lPAR and rPAR) and sensory cortices (postcentral gyrus, lS1 and rS1). All ROIs were relabeled from left and right to affected and unaffected, based on lesion location. For the sake of comparison, left ROIs were labeled affected for the HCs. Statistical analysis included univariate analysis to compare between groups (HC and Stroke Baseline/Stroke Post Training) and estimate the effect sizes. Linear regressions were used to compare CMC to clinical measurements in Stroke group. We did not statistically account for multiple comparisons in this paper, because it is an exploratory project with low sample size. So, we estimated effect size for each comparison to demonstrate whether the effect size of difference between groups and session is low, medium, or high.

## III. Results

### A. EEG Power During Standing

**Figure 1.**
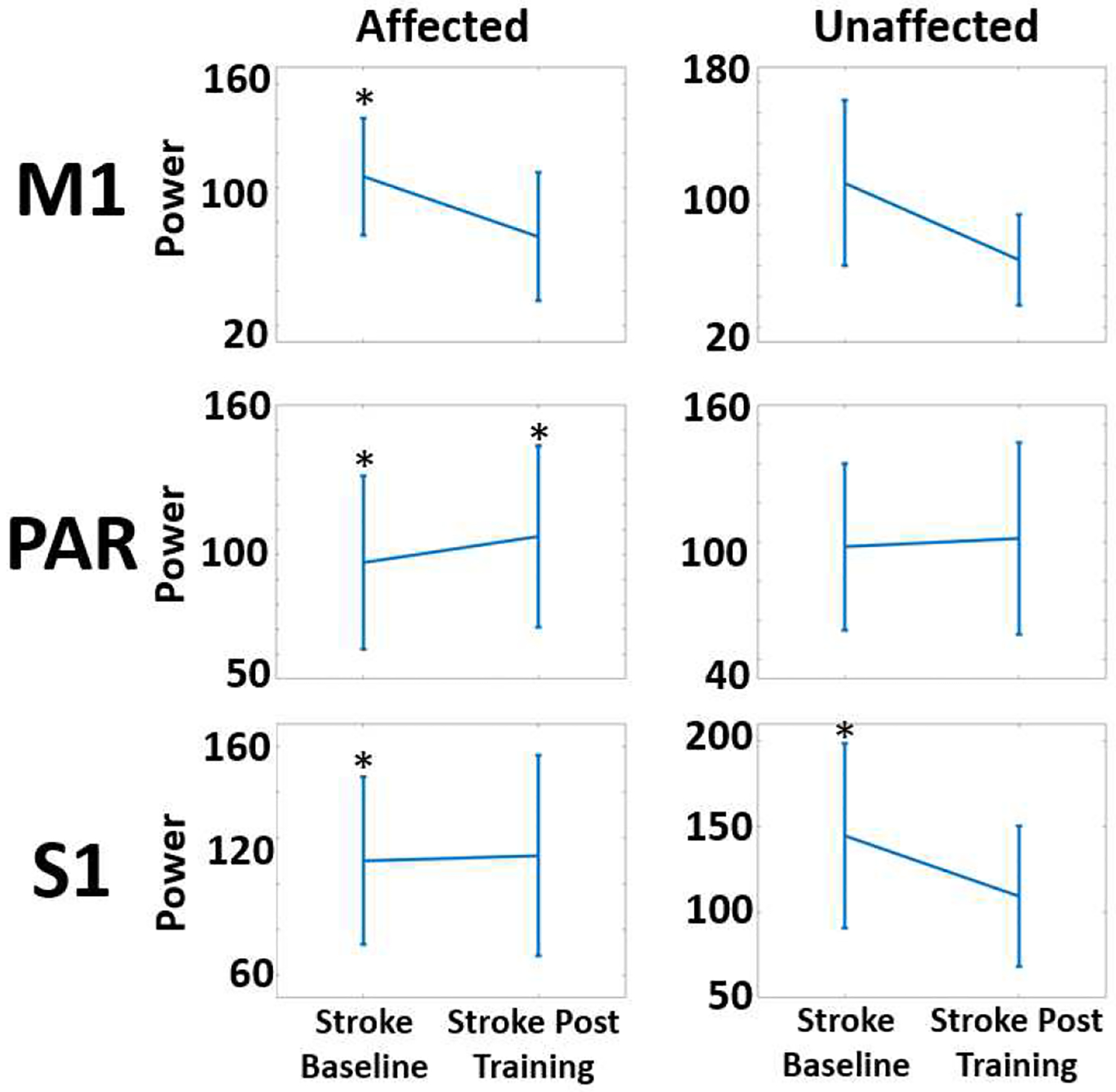
Percent Difference in EEG power of selected regions of interest (primary motor cortex M1, parietal cortex PAR, and primary sensory cortex (S1) in the Affected and Unaffected hemispheres of stroke group relative to HC group Left and Right hemispheres.

Results demonstrate higher EEG power in cortical sources localized in the motor, parietal, and sensory cortices stroke group at baseline (pre-training) testing session in comparison to HC group. EEG power of motor cortices post training in the stroke group renormalized towards more similarity with the HC group, while it stayed at high levels in the parietal and sensory regions. As shown in table I and figure 1, the univariate analysis consistently showed moderate effect size of difference between HC group and stroke group at baseline (pre-training), driven by higher EEG power in the stroke group.

### B. Corticomuscular Connectivity During Standing

**Figure 2.**
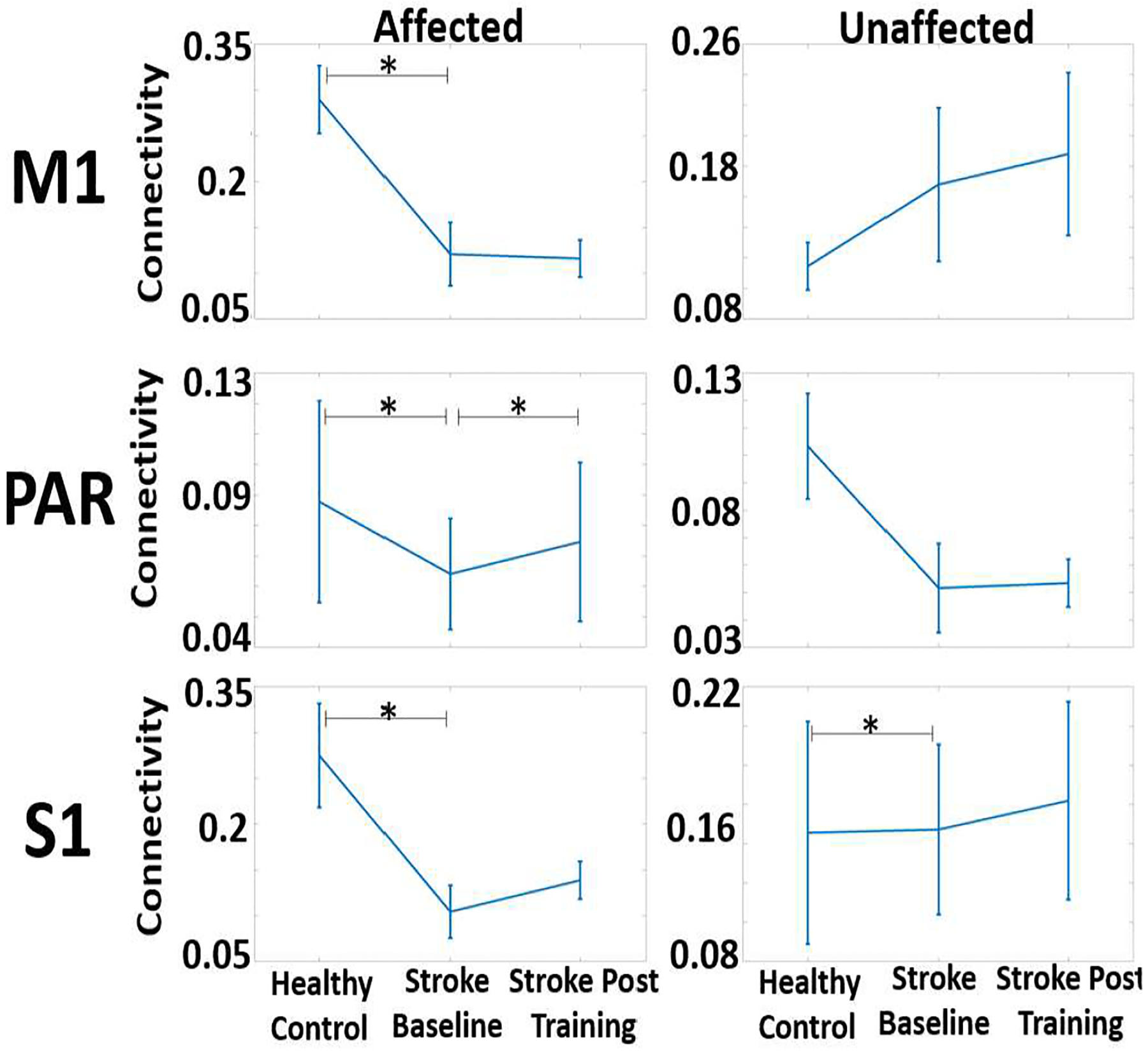
Directional connectivity from M1, PAR and S1 to contralateral lower extremity muscles.

Table 2 and Figure 2 compare the directional connectivity (nDTF measure) during standing, from sensorimotor regions to contralateral lower extremity muscle, for both HC and stroke participants. The data show lower CMC from affected regions to contralateral muscles (see Figure 2) during baseline, with a trend toward renormalization to HC group values at post-training for affected parietal and sensory cortices. Connectivity from unaffected M1 to contralateral muscles is higher in stroke group at baseline and post training, suggesting a compensation effect.

### C. CMC and its Relationship with Clinical Measures in Stroke

**Figure 3.**
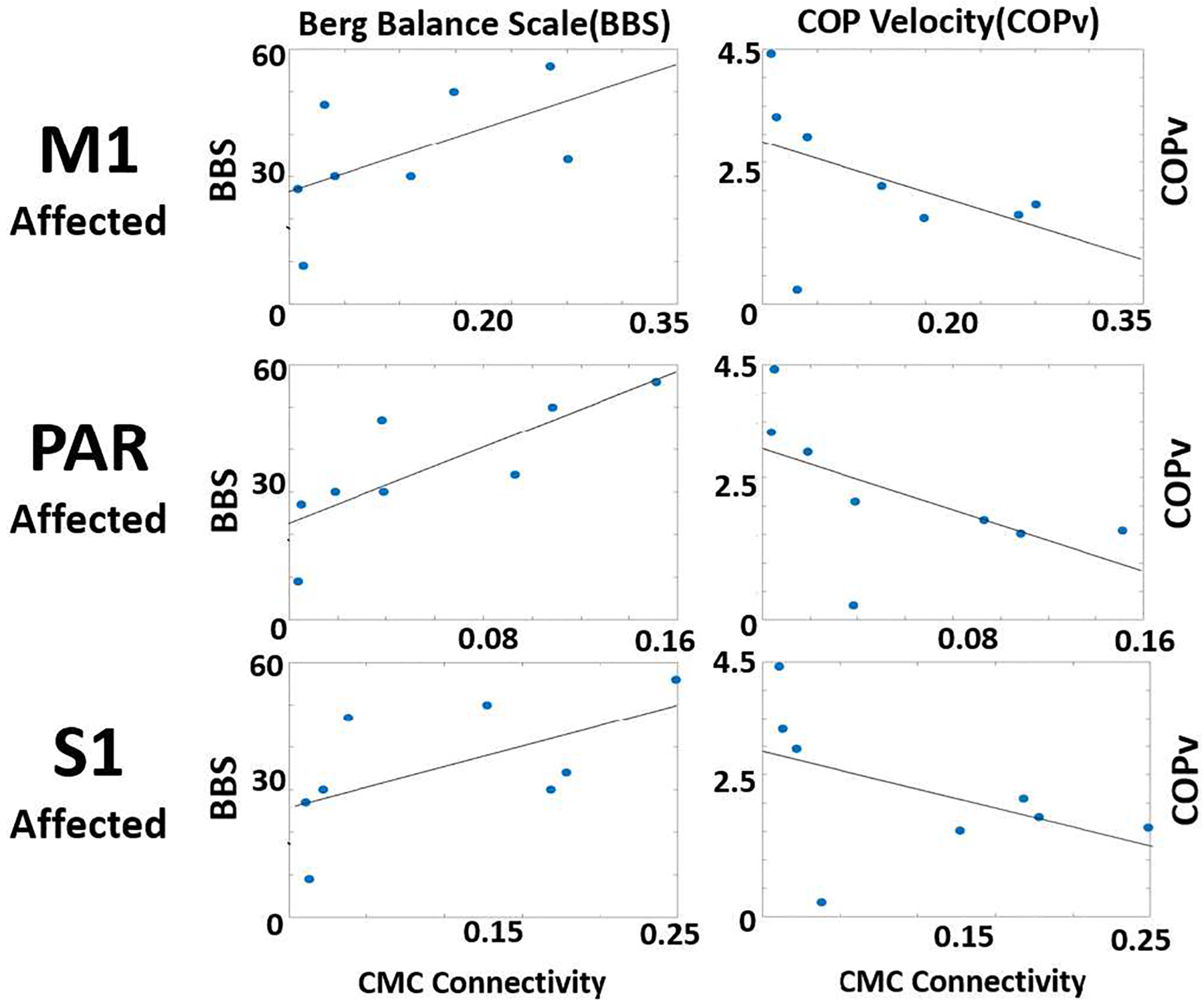
Relationship between CMC and assessments of balance (BBS vs CMC in the left column, and COP velocity vs CMC in the right column) in Stroke population

To explore the relationship between CMC and functional mobility, linear regression analysis was performed looking into the relationship between CMC measures from the selected sensorimotor regions (M1, PAR and S1) to contralateral lower extremity muscles during standing and two different measures of balance (Berg Balance Score, BBS, and COP velocity with eyes closed during perturbation task performed on the Movendo balance platform). As shown in Figure 3 and Table 3, higher CMC in M1, PAR, and S1 correlates with higher (better) BBS and lower (better) COP velocity for all ROIs, and the relationship between CMC from PAR Affected to contralateral muscles and BBS is statistically significant. The regression analysis results for the other ROI are not statistically significant but have moderate R values and effect sizes.

## IV. Discussion

The preliminary data in this paper show a trend of higher EEG power during stranding in the stroke group and lower connectivity from affected hemisphere sensorimotor cortices to contralateral lower extremity muscles. To our knowledge this is the first examination of corticomuscular connectivity between sensorimotor cortices and peripheral muscles during standing in subacute stroke participants.

The higher EEG power and lower CMC during standing in stroke subjects suggest higher cognitive engagement but less efficiency in controlling peripheral muscles [10]. The higher activation in EEG power during standing suggests the need to recruit more cortical resources to maintain balance during standing in subacute stroke. Post training, which is also around 6 months post stroke, EEG power decreases relative to baseline but remains higher than HC group data, showing a trend of normalization toward HC data. CMC data show no re-normalization, where lower CMC in the affected hemisphere remains low post training. Interestingly lower CMC correlates with worse balance performance, showing an important role of sensorimotor cortices connectivity with peripheral muscles to maintain balance, and a deficit in this mechanism post stroke.

The data in this paper are preliminary and they cannot confirm any conclusion regarding cortical control of standing post stroke, but the effect sizes show a reorganization in cortico-muscular connectivity during standing post stroke and support the importance to continue exploring this research in larger sample size. By gaining a deeper understanding of the factors contributing to post-stroke standing impairments, including change in corticomuscular connectivity, healthcare professionals can provide more effective and targeted care by developing target rehabilitation interventions to enhance the recovery of standing and balance performance and well-being of stroke survivors, and improve motor function and quality of life for stroke survivors.

## Figures and Tables

**Table 1.** EEG Power During Standing, Beta Band(12–30 Hz) Table 1 – unpaired t-test comparisons between the HC and stroke group (at baseline and post training timepoints).

ROI	Comparison	P	Cohen’s D
Affected M1	HC vs Stroke Baseline	0.020	−1.193
Affected M1	HC vs Stroke Post Training	0.131	−0.942
Unaffected M1	HC vs Stroke Baseline	0.081	−0.812
Unaffected M1	HC vs Stroke Post Training	0.131	−0.995
Affected PAR	HCvs Stroke Baseline	0.031	−1.066
Affected PAR	HC vs Stroke Post Training	0.032	−1.460
Unaffected PAR	HC vs Stroke Baseline	0.060	−0.884
Unaffected PAR	HC vs Stroke Post Training	0.097	−1.045
Affected S1	HC vs Stroke Baseline	0.021	−1.151
Affected S1	HC vs Stroke Post Training	0.052	−1.285
Unaffected S1	HC vs Stroke Baseline	0.034	−1.033
Unaffected S1	HC vs Stroke Post Training	0.054	−1.283

**Table 2.** CMC During Standing, Beta Band(12–30Hz) Table 1. Unpaired T-test comparison between groups. Stroke data is compared to HC group at baseline and post-training timepoints,

ROI	Comparison	P	Cohen’s D
Affected M1 to Contralateral EMG	HC vs Stroke Baseline	0.010	1.748
Affected M1 to Contralateral EMG	HC vs Stroke Post Training	0.010	2.892
Unaffected M1 to Contralateral EMG	HC vs Stroke Baseline	0.335	−0.413
Unaffected M1 to Contralateral EMG	HC vs Stroke Post Training	0.236	−0.700
Affected PAR to Contralateral EMG	HC vs Stroke Baseline	0.558	0.408
Affected PAR to Contralateral EMG	HC vs Stroke Post Training	0.765	0.203
Unaffected PAR to Contralateral EMG	HC vs Stroke Baseline	0.077	1.121
Unaffected PAR to Contralateral EMG	HC vs Stroke Post Training	0.073	1.725
Affected S1 to Contralateral EMG	HC vs Stroke Baseline	0.047	1.802
Affected S1 to Contralateral EMG	HC vs Stroke Post Training	0.090	1.703
Unaffected S1 to Contralateral EMG	HC vs Stroke Baseline	0.982	−0.013
Unaffected S1 to Contralateral EMG	HC vs Stroke Post Training	0.836	−0.136

**Table 3.** CMC Baseline Regressions, Beta Band(12–30 Hz) Table 3 – unpaired t-test comparisons CMC and clinical measures (Berg Balance Scale and COP velocity).

ROI	Clinical Measure	P	R
Affected Ml to Contralateral EMG	Berg Balance Scale	0.148	0.561
Affected Ml to Contralateral EMG	COP Velocity	0.259	−0.454
Affected PAR to Contralateral EMG	Berg Balance Scale	0.017	0.799
Affected PAR to Contralateral EMG	COP Velocity	0.143	−.566
Affected S1 to Contralateral EMG	Berg Balance Scale	0.124	0.590
Affected S1 to Contralateral EMG	COP Velocity	0.240	−0.470

## Clinical Relevance—

This study evaluates corticomuscular connectivity during standing in healthy controls and individuals with subacute stroke (within 40 days of injury). Better understanding of cortical control of standing post stroke is important to improve strategies used in mobility rehabilitation.
